# Time-dependent effect of prone position in ARDS: considerations for future research

**DOI:** 10.1186/s13054-024-04915-1

**Published:** 2024-04-18

**Authors:** Yuxian Wang, Ming Zhong

**Affiliations:** 1 Department of Critical Care Medicine, Zhongshan Hospital of Fudan University, Shanghai, 200032 China; 2 Shanghai Institute of Infectious Disease and Biosecurity, School of Public Health, Fudan University, Shanghai, China; 3 Shanghai Key Laboratory of Lung Inflammation and Injury, Shanghai, China

To the Editor

We have read the article by Yuan et al. [1] with great interest, where they indicated that prone position significantly reduced ventilation/perfusion (V/Q) mismatch in patients with early ARDS, while it increased V/Q mismatch in persistent ARDS patients. However, there are several factors that might influence the reported findings:

Firstly, it is important to consider that the effect of prone position is time-dependent. Instead of defining a 20% improvement in PaO_2_/FiO_2_ at 2 h as a responder, it would be more appropriate to compare PaO_2_/FiO_2_ at the end of the prone position to the baseline. PP has a timely gravity effect on the change of ventilation, and also takes time to gradually open up collapsed or consolidated alveoli and drainage secretion. Additionally, previous studies have shown that PaO_2_/FiO_2_ increases during the first 3 h of PP, but further improvement in PaO_2_/FiO_2_ occurs in later stages due to increased ventilation and blood flow then more matched regions [2]. Therefore, we believe that using the PaO_2_/FiO_2_ at 2 h in the PP may not accurately define the reactivity of PP at 12 h. In the study, out of the 24 patients with persistent ARDS, 23 (95.8%) had moderate ARDS. Although the Shunt-EIT% (including ventral and dorsal) and Total unmatched units increased significantly, there was no statistical difference in the PaO_2_/FiO_2_ of these patients after 12-h PP (Table 2). There is a lack of information on changes in matching or oxygenation during the 12-h period. It would be valuable to understand whether there is a peak effect of PP and its timing, as this could provide guidance for implementing this intervention. Therefore, further investigations which assess the time-dependent effect of PP in ARDS of different severity and time stage are warranted.

Secondly, this study defined ARDS days as starting from the initiation of non-invasive or invasive ventilation until the first PP. The initiation of non-invasive or invasive ventilation alone cannot be classified as ARDS without the support of imaging results at the time and the exclusion of heart failure. Figure E2 illustrates that the initial PaO_2_/FiO_2_ values were around 200 mmHg during the first two days of non-invasive/invasive use but decreased thereafter with a downward trend. There might have been a delay for intubation or PP in some persistent ARDS cases where it took seven days before initiating PP. Therefore, it is possible that the effectiveness of PP is not solely attributed to persistent ARDS, but also influenced by underlying pathological mechanisms, such as excessive respiratory drive leading to patient self-inflicted lung injury (P-SILI) [3, 4] or VILI [5], and aggravated basic disease. Moreover, the impact of early PP on the progression of ARDS necessitates further investigation.

Considering an early attempt at awake PP might also be reasonable. For persistent ARDS cases with suboptimal improvements in oxygenation despite PP, it is worth considering alternative approaches. Could a extended duration of PP or combining PP with inhaled nitric oxide (iNO) yield better results? Further research is needed to address these questions and explore how it can lead to less shunting and facilitate efficient gas exchange.

## Data Availability

Not applicable.

## Abbreviations

ARDS: Acute respiratory distress syndrome
V/Q: Ventilation/perfusion
PP: Prone position
EIT: Electrical impedance tomography
P-SILI: Patient self-inflicted lung injury
iNO: Inhaled nitric oxide
